# Emerging evidence linking the gut microbiome to neurologic disorders

**DOI:** 10.1186/s13073-018-0609-3

**Published:** 2018-12-21

**Authors:** Jessica A. Griffiths, Sarkis K. Mazmanian

**Affiliations:** Division of Biology and Biological Engineering, California Institute of Technology, East California Boulevard, Pasadena, CA 91125 USA

## Abstract

The gut microbiome contributes to the development and function of the immune, metabolic, and nervous systems. Furthermore, commensal bacteria modulate symptoms and pathology in mouse models of neuropsychiatric and neurodevelopmental diseases. Uncovering mechanisms that are utilized by the microbiome to mediate gut–brain connections may provide novel opportunities to target therapies to the gut in order to treat neurologic disorders.

## The gut microbiome and brain disorders

Disorders of the brain such as anxiety, depression, epilepsy, and autism spectrum disorder (ASD) have been linked to the gut microbiome, largely in preclinical models [1]. Microbiome changes in humans have been cataloged in many neurologic conditions, and mouse models have revealed that gut microbes contribute to disease progression and severity of symptoms [1]. Recent work in this area reports new findings in disorders of the brain and in well-established murine models of behavior [2–5]. These studies indicate that a combination of factors, including prenatal environments, diet, and host genetics, impact the fitness of an individual’s microbiome. Changes to host microbiome composition coincide with neurologic changes affecting behavior, neurotransmitter levels, stress response, and gene expression in the brain [2–5]. These findings highlight a growing appreciation that gut bacteria may contribute to neuropsychiatric disorders, and potentially reveal attractive targets for translational studies in humans.

## Early-life influences

Upon birth, the sterile gut of a newborn is colonized by microbiota, which are required for normal adolescent brain and immune system development [6]. These early colonizers are instrumental during development in educating the immune system, metabolizing nutrients, and influencing complex behaviors. One example of the impact of early colonization is illustrated by differences in cognitive scores between infants born via cesarean section and vaginally born children [6]. The lower scores of children born via cesarean section may result from differences in early gut colonization by microbes from the mother’s skin (caesarean) or vagina (natural birth), respectively [6]. Infants who have increased exposure to antibiotics have a greater risk of developing behavioral symptoms such as depression [1]. Further evidence of early-life influences comes from observations that formula feeding, which alters the microbiome, may be a risk factor for ASD [7], although considerable additional work in humans is needed to establish causality.

Prenatal stress can also affect microbiome composition after birth and is linked to increased risk of developmental disorders of the brain [2]. Stress induces distinct changes in the vaginal microbiome composition, so mothers who are under stress may transmit an imbalanced bacterial community to their offspring [2]. Stress responses can be measured by activation of the hypothalamic-pituitary-adrenal (HPA) axis, which is one of the major routes of communication between the periphery and the brain. Male mouse pups born to stress-exposed dams exhibit increased stress, indicated by elevated levels of corticosterone in the serum and altered gene expression in the hypothalamus [2]. It is difficult to determine whether this phenotype results from prenatal effects or microbial colonization at birth. Jašarević et al. [2] showed that colonization of mice with untreated, control vaginal microbiota normalized, albeit partially, features such as body weight and corticosterone levels following acute stress in male offspring. The fact that these negative effects could not be reversed fully suggests that stress also affects the mouse pups before birth (i.e., prior to microbiome exposure). This idea is supported by transcriptomic data from the murine fetal intestine that reveals differential expression of genes implicated in innate immunity and inflammation based on prenatal stress-exposure in males [2]. Therefore, it is likely that environmental risk factors, such as prenatal stress, alter the intestinal niche of the fetus before microbes even colonize the gut and could select against beneficial microbes.

In theory, reduced prenatal stress should promote microbiome health and normal immune system development. When early-life perturbations such as cesarean section or treatment with antibiotics contribute to symptoms, colonizing newborns with missing or depleted microbes or with a complex microbiome from a verified healthy donor may provide benefits [8].

## Diet-induced changes

Although the initial colonization of the gut plays a critical role in shaping the microbiome, diet has a significant impact on microbial composition throughout life [1, 3]. A high-fat diet (HFD) can result in obesity by inducing gut dysbiosis [3]. Although obesity and diabetes are not traditionally considered neurologic disorders, they often co-occur with anxiety and depression [3].

Diet-induced obesity (DIO) mice fed an HFD exhibit hallmark characteristics of diabetes, including insulin resistance and hyperglycemia, and also display behaviors symptomatic of anxiety and depression [3]. Soto et al. [3] found that DIO mice display abnormal neurotransmitter levels including increases in brain levels of γ-aminobutyric acid (GABA) and tryptophan, a precursor of serotonin, which are associated with mood and behavior in humans. Antibiotic treatment with vancomycin and metronidazole depletes Gram-positive and Gram-negative anaerobic bacteria in the gut, respectively. Both antibiotic treatments ameliorated the behavioral deficits and diabetes-like symptoms found in DIO mice. This evidence indicates that an HFD may enrich populations of gut microbes that play a role in the physiology of obesity and diabetes, and suggests that treatments that eliminate certain microbes may help treat both metabolic and behavioral conditions [3].

Conversely, some diets have therapeutic potential for neurologic disorders. The ketogenic diet (KD) consists of high fat content foods but minimal amounts of carbohydrates, which causes metabolism of fat instead of carbohydrates for energy. The KD has been used to treat epilepsy for a century, but the importance of the gut microbiome in mediating this effect was largely unknown [4]. Olson et al. [4] found that administration of the KD to a mouse model of epilepsy that uses electrical stimulation to induce seizures (6-Hz seizure mouse model) resulted in changes in the microbiome composition and made the mice more resistant to seizures. The microbiome is necessary for the beneficial effects of the diet, as antibiotic-treated and germ-free mice fed the KD do not reap the protective effects of the diet. KD-fed mice are enriched in the bacteria *Akkermansia muciniphila*, *Parabacteroides merdae*, and *Parabacteroides distasonis*, which were shown to be involved in promoting the diet’s anti-seizure effects. Manipulation of the gut microbiome through the KD or colonization with *A. mucinophilia* and *Parabacteroides* provided protective benefits against seizures by altering brain neurotransmitter levels, including GABA and glutamate in the hippocampus [4]. GABA is the major inhibitory neurotransmitter in the brain, and reduced levels are known to exacerbate seizures. Diet is thus an effective means of manipulating neurotransmitter levels in the brain, with the resulting diet–microbiome interactions mediating the effects of seizures.

## Genetic interactions

Studies have begun to use DNA sequencing to investigate the impact of host genetics on the microbiome and to look at how gene–environment interactions affect neurological disease [9]. Whole-genome association studies have revealed genetic variants involved in host immunity and metabolism that may predispose individuals to gut dysbiosis [9]. Genetic mutations that are associated with neurological disorders may also alter the host intestinal niche and perturb the microbiome.

ASD is a complex neurological disorder with diverse genetic and environmental etiologies [5]. Children with ASD are at least three times more likely to experience chronic gastrointestinal symptoms than neurotypical children, suggesting that ASD physiology is linked to gut dysbiosis [10]. Accordingly, a number of studies have shown that the microbiome is altered in children with ASD compared with controls [5]. Many genetic variants associated with ASD involve synaptic transmission [5]. A mutation affecting SHANK3, a scaffolding protein in the postsynaptic density of excitatory neurons, contributes to about 2% of ASD cases in humans [5]. A mouse line with homozygous knockout of *Shank3* exhibited repetitive behaviors and abnormal social interactions, which are characteristic features of human ASD. Tabouy et al. [5] showed that *Shank3*^*−/−*^ mice have reduced gut microbiome diversity, with diminished populations of certain species of bacteria, such as *Lactobacillus reuteri*, *Lactobacillus brevis*, and *Lactobacillus ruminis*. Gut colonization with *L. reuteri* improved behavioral outcomes in male mice and increased the expression of GABA receptors in the brain [5]. This finding is consistent with the abnormal excitatory and inhibitory synaptic transmission through glutamate and GABA signaling reported in ASD [11]. It appears that enrichment of (or treatment with) specific commensal microbes may be a promising avenue for ameliorating certain behavioral disorders.

## Future directions

At present, genetic and environmental factors (and their effect on the microbiome) are investigated separately. The effects of genetic predispositions on neurological disorders are compounded by diet choices, prescription medications, exercise, age, and life experiences, all of which shape the microbiome [2]. Studying these components individually ignores critical interactions between various factors, thus limiting our understanding of the complex mechanisms linking gut dysbiosis and neurologic conditions. Preclinical models that can rigorously control for and test genetic and environmental factors will serve as useful representations of the diverse influences that impact neurologic function, including the microbiome. Animal models provide opportunities to discover therapeutic options, such as microbiota transplants and potential dietary interventions, that can be individually tailored for distinct neuropsychiatric and neurodevelopmental disorders. Microbiome-based treatments aimed at influencing neurological responses, such as neurotransmitter release, stress responses, and neurological development, could be designed in accordance with an individual’s genetic risk for a given disease. As it remains challenging to correct genetic predispositions, the correction of altered microbiomes appears to be a more viable approach toward novel therapeutics for neurologic disorders.

## Abbreviations

ASD: Autism spectrum disorder
DIO: Diet-induced obesity
GABA: γ-Aminobutyric acid
HFD: High fat diet
KD: Ketogenic diet
